# Editorial: Modulators of Skeletal Muscle Hypertrophy: Mechanisms to Lifestyle Strategies

**DOI:** 10.3389/fphys.2022.893698

**Published:** 2022-04-26

**Authors:** Vandré C. Figueiredo, Llion A. Roberts, David Cameron-Smith, James F. Markworth

**Affiliations:** ^1^ Center of Muscle Biology, University of Kentucky, Lexington, KY, United States; ^2^ School of Health Sciences and Social Work, Griffith University, Gold Coast, QLD, Australia; ^3^ School of Human Movement and Nutrition Sciences, University of Queensland, Brisbane, QLD, Australia; ^4^ Singapore Institute for Clinical Sciences, Singapore, Singapore; ^5^ Department of Animal Sciences, Purdue University, West Lafayette, IN, United States; ^6^ Center on Aging and the Life Course, Purdue University, West Lafayette, IN, United States

**Keywords:** skeletal muscle, hypertrophy, atrophy, satellite cell, protein synthesis, protein degradation

## Introduction

The central importance of skeletal muscle mass and strength as key determinants of overall health, longevity, disease survival, functional independence, and quality of life is now well established. Understanding the basic molecular and cellular mechanisms that regulate skeletal muscle hypertrophy, as well as the effect of various pharmacological and non-pharmacological (including lifestyle) strategies to build muscle mass may ultimately lead to better interventions to maintain or improve muscle size and strength across the life span. For this reason, we focused on the “*Modulators of skeletal muscle hypertrophy: Mechanisms to Lifestyle Strategies*” as a Research Topic of interest. In this Frontiers Research Topic, authors from around the globe have tackled unique aspects of these major challenges, providing cutting edge original research studies and comprehensive review articles.

### Of Mice and (wo)men

Two investigations in this Research Topic utilized genetically modified mouse models to better understand the underlying mechanisms of postnatal muscle growth. Zeng and colleagues tested the hypothesis that the muscle fiber type transition (from slow to fast) which normally occurs in early life is required for muscle hypertrophy (Zeng et al.). The authors knocked out the *Myh4* gene (encoding the murine fast-twitch myosin heavy chain IIb protein) and demonstrated impaired postnatal growth of the gastrocnemius muscle during the transition from slow to fast fiber phenotype. By overexpressing PGC-1α or feeding mice the AMPK agonist AICAR, the authors showed that forcing a more oxidative fiber type profile could partially protect against muscle loss resulting from the ablation of the myosin heavy chain IIb protein. On the other hand, traditional genetic (myostatin deficiency) and pharmacological (clenbuterol treatment) stimulators of muscle hypertrophy had little impact on muscle of mice lacking a functional *Myh4* gene. These results suggest that promoting oxidative metabolism may be a therapeutic strategy to mitigate muscle loss in conditions where glycolytic myofibers are preferentially affected such as age-associated muscle wasting (sarcopenia).

Barbé and colleagues identified the *Pak1* gene (encoding the p21-activated kinase 1 protein) as a potential mediator of the hypertrophic actions of myostatin deficiency on skeletal muscle from various transcriptomic datasets using different experimental strategies to inhibit myostatin activity (Barbe et al.). Local expression of *Pak1* was shown to be markedly increased in response to myostatin inhibition and positively associated with ensuing muscle hypertrophy in these mouse models. However, genetically deleting the *Pak1* gene did not affect muscle hypertrophy in response to myostatin inhibition. Given the existence of different members of the PAK protein family, in particular PAK2, it is possible that some redundancy exists regarding muscle metabolism between PAK1 and PAK2, given that simultaneous deletion of both genes was previously shown to result in muscle atrophy (Joseph et al., 2017).

Resistance training promotes skeletal muscle growth by myofiber hypertrophy. However, the underlying mechanisms are not fully understood in humans yet. Resident muscle stem cells termed satellite cells are often cited as a main mechanism of myofiber hypertrophy in response to resistance training *via* their unique ability to fuse with post-mitotic muscle cells, thereby resulting in myonuclear accretion. Shamim and colleagues investigated whether the addition of endurance exercise to a resistance exercise training program might interfere with muscle hypertrophy, an important consideration for all individuals who partake in mixed modality concurrent exercise training programs (Shamim et al.; Shamim et al.). The authors demonstrated that the size of fast-twitch (type II) myofibers but not slow-twitch (type I) myofibers increased in active male participants that performed 12-weeks of either endurance, resistance, or alternate day concurrent training. Interestingly no changes were observed in satellite cell content in either fiber type, and significant changes in myonuclear number were only observed in type I, but not type II myofibers. Nonetheless, the authors noted that the magnitude of increase in fiber type specific cross-sectional area was positively correlated with the changes in myonuclear number in both fiber types. This data adds to the growing body of evidence questioning whether or not myonuclear accretion *via* satellite cell fusion is a prerequisite to changes in myofiber hypertrophy in humans.

Talking about questioning preexisting paradigms, Fox and colleagues investigated whether frequent manipulation of resistance training variables in human volunteers might result in different adaptations to the myofibrillar or sarcoplasmic protein pools in skeletal muscle (Fox et al.). The potential for preferential hypertrophy of myofibrillar or sarcoplasmic cellular fractions of skeletal muscle cells has been a topic of enthusiastic debate in recent years (Roberts et al.). In this thought-provoking investigation, Fox et al. provide data to corroborate the notion that different resistance exercise training protocols might differentially affect myofibrillar and cytosolic compartments in resistance trained men. An increased myofibrillar spacing i.e., the non-contractile portion of muscle cell, seems to be associated with 8-weeks of a higher volume-training load, at least in muscle biopsy samples obtained 4 days following the last bout of resistance exercise. Whether these adaptations persist or are resolved warrants further investigation. Nevertheless, the study by Fox et al. highlights that myofiber hypertrophy induced by resistance training may partly occur due to expansion of the cytosolic subcellular fraction.

Dam and colleagues aimed to determine whether transdermal estrogen therapy might improve muscle adaptation to resistance exercise in postmenopausal women, given that the decline in estrogen levels is accompanied with loss of muscle mass at this period (Dam et al.). In this double-blinded randomized controlled study, the authors demonstrated that the group of postmenopausal women that received transdermal estrogen therapy displayed significantly greater increases in muscle cross-sectional area, whole-body fat-free mass, and handgrip strength compared to the control group following 12 weeks of resistance exercise training. These clinically relevant results show that transdermal estrogen therapy leading to increased circulating estradiol levels can greatly benefit postmenopausal women during resistance training.

Finally, Viecelli and Aguayo discuss the state-of-the-art of the research of resistance exercise variables, including volume, intensity, rest interval, frequency, range of motion, and muscle failure, on muscle hypertrophy and muscular strength (Viecelli and Aguayo). In this detailed review of the literature, Viecelli and Aguayo summarize the latest evidence on the resistance training variables affecting muscle adaptation relevant to the general population, with important directions to training prescription.

## Concluding Remarks

Knowledge of the adaptations to resistance training and the lifestyle interventions that affect such responses are of utmost importance to create and apply novel strategies to improve overall health as the population in most countries ages. The studies in this Research Topic not only provide new important data to improve our current understanding of skeletal muscle physiology, but also poses new questions and provokes reanalysis of previously established paradigms. Whether myonuclear accretion resulting from satellite cell fusion is important for muscle hypertrophy and whether sarcoplasmic hypertrophy is a true physiological adaptation to resistance training will still be debated, but the studies presented herein and future work they stimulate will help solve these puzzles. As demonstrated in this Research Topic, mechanistic investigations in pre-clinical mouse models will continue to be invaluable in improving our understanding of the basic mechanisms regulating skeletal muscle hypertrophy. However equally important are clinically relevant human trials which bring the basic science of muscle biology from bench-to-bedside and will ultimately help health practitioners and physicians chose the best interventions to promote muscle hypertrophy during resistance training throughout the life course. We, the editors, are extremely pleased to see the investigations published in this Frontiers Research Topic, the relevance that they will have to our understanding to the field of skeletal muscle physiology, and excited by the groundwork the empirical studies have laid for further research investigations to build upon.
